# Object Detection Based on Faster R-CNN Algorithm with Skip Pooling and Fusion of Contextual Information

**DOI:** 10.3390/s20195490

**Published:** 2020-09-25

**Authors:** Yi Xiao, Xinqing Wang, Peng Zhang, Fanjie Meng, Faming Shao

**Affiliations:** Department of Mechanical Engineering, College of Field Engineering, Army Engineering University of PLA, Nanjing 210007, China; xiao_yi0908@163.com (Y.X.); ZPhlgfs19951027@163.com (P.Z.); beilimeng1992@163.com (F.M.); shaofaming@163.com (F.S.)

**Keywords:** object detection, Faster R-CNN, context, skip pooling, guided anchor RPN

## Abstract

Deep learning is currently the mainstream method of object detection. Faster region-based convolutional neural network (Faster R-CNN) has a pivotal position in deep learning. It has impressive detection effects in ordinary scenes. However, under special conditions, there can still be unsatisfactory detection performance, such as the object having problems like occlusion, deformation, or small size. This paper proposes a novel and improved algorithm based on the Faster R-CNN framework combined with the Faster R-CNN algorithm with skip pooling and fusion of contextual information. This algorithm can improve the detection performance under special conditions on the basis of Faster R-CNN. The improvement mainly has three parts: The first part adds a context information feature extraction model after the conv5_3 of the convolutional layer; the second part adds skip pooling so that the former can fully obtain the contextual information of the object, especially for situations where the object is occluded and deformed; and the third part replaces the region proposal network (RPN) with a more efficient guided anchor RPN (GA-RPN), which can maintain the recall rate while improving the detection performance. The latter can obtain more detailed information from different feature layers of the deep neural network algorithm, and is especially aimed at scenes with small objects. Compared with Faster R-CNN, you only look once series (such as: YOLOv3), single shot detector (such as: SSD512), and other object detection algorithms, the algorithm proposed in this paper has an average improvement of 6.857% on the mean average precision (mAP) evaluation index while maintaining a certain recall rate. This strongly proves that the proposed method has higher detection rate and detection efficiency in this case.

## 1. Introduction

With the vigorous development of deep learning, object detection technology has received extensive attention and many scholars have conducted in-depth research. Object detection algorithms include frame difference [1], background subtraction [2], optical flow [3], and Hough transform [4] methods. These are commonly used as traditional object detection methods, and they have many limitations in the process of detecting objects [5]; for example, the classification is too narrow, the application scenarios are limited to simple backgrounds, too much manual intervention is required to obtain features, or autonomy cannot be achieved. They also have serious shortcomings in robustness, which leads to problems such as poor generalization ability and poor detection results. Traditional object detection algorithms can no longer meet the application requirements of industrial and military fields, and object detection based on deep learning has thus become a popular research direction for many scholars around the world.

Object detection algorithms based on deep learning are basically not restricted by the application scenario; they can realize autonomous learning and have good detection performance [6]. At present, object detection algorithms based on deep learning tend to be relatively mature and can have better detection performance in specific scenes, such as pedestrian detection [7], face detection [8], etc. These algorithms can be widely used in intelligent monitoring systems [9], intelligent transportation systems [10], military object detection [11], medical object detection [12], etc. However, there is more room for optimization in more special scenes [13], such as the problems of occlusion, too small scale, deformation, and camouflage of the object in the image. For example, problems exist in how the object can be accurately and quickly captured by part of the feature information when it is partially occluded and how it can be selected and located from the limited feature information when its scale is too small.

It is also worth mentioning the problem of generating more suitable anchors in object detection. An anchor is often used as a benchmark frame for classification and regression in object detection. Most of the commonly used anchoring methods cannot cover the object area well due to their fixed shape. In order to ensure a high recall rate, an excessively large number of anchors is required, which will result in too many negative samples in the range enclosed by the anchors. The general anchor generation method will have such problems, resulting in poor detection performance.

In order to deal with the above problems, this paper proposes an improved algorithm based on faster region-based convolutional neural network (R-CNN), with higher detection performance. It uses the guided anchor method [14] to replace the previous region proposal network (RPN), selective search [15], and other candidate region methods. On this basis, the context feature is also used to obtain the network module [16] to solve the problem of object occlusion. The skip pooling method [17] is used for multi-feature fusion of different deep neural network algorithms to solve the problem of small object scale, so as to optimize the detection performance of Faster R-CNN in complex scenes.

This paper proposes a Faster R-CNN algorithm combined with skip pooling and fusion of contextual information. This method can provide detection and classification accuracy comparable to the latest methods. The main contributions of this paper are summarized as follows:

- A new RPN algorithm is used, guided anchor RPN (GA-RPN), which predicts the position and shape of the anchor to generate a sparse anchor that can tightly surround the object and fit the object shape. This method can reduce the calculation time while maintaining a high recall rate.
- A context information feature extraction network fused with contextual information is used. The network can provide the contextual information of a partially occluded object for the proposed method and use the contextual information around the object as the information reference for the object’s own feature detection.
- In order to solve the problem of less feature information from smaller objects in a single feature layer, this paper proposes skip pooling, which combines the features of multiple feature layers, greatly improving the expression ability of features, and is suitable for small object detection.

The organization of the rest of this paper is as follows: Section 2 is about related work. Section 3 is a detailed discussion of the proposed method, which includes two parts, the context feature extracted model, and the multi-layer feature fusion. Section 4 analyzes the experimental results. Finally, conclusions and future work are presented in Section 5.

## 2. Related Work

Object detection algorithms are mainly traditional or based on deep learning. Currently, algorithms based on deep learning are widely used in various fields as a mainstream method of object detection. In the process of object detection, there can be many uncertain factors, such as too-small object scale, rotated object, etc. In addition, the object is usually affected by factors such as illumination and occlusion, which makes the detection algorithm difficult. In order to improve the generalization ability of the algorithm, researchers need to make appropriate modifications and improvements according to the specific situation.

For example, for the object rotation problem, a simple but effective method was proposed in [18] to train rotation-invariant and Fisher discriminative CNN models to further boost object detection performance. For the object occlusion problem, a simple but effective framework was developed in [19] for camouflaged object detection (COD), termed search identification network, which can help facilitate future research on COD. This paper focuses on the problem of too-small object scale and partial occlusion. The network framework of object detection affects the detection process. The main purpose of optimization for this framework is to avoid redundant and missed detection. Therefore, the framework structure usually determines the detection performance. The current object detection algorithm framework based on deep learning includes a two-stage algorithm represented by the R-CNN series and the one-stage algorithm represented by you only look once (YOLO) and single shot detector (SSD) series. The main difference between the two is that the two-stage algorithm needs to generate a large number of preselected boxes that may contain objects, and then perform fine-grained object detection. The object detection framework requires a preselected box algorithm.

The pioneering work of the two-stage algorithm for object detection began with R-CNN [20], which includes three modules: region proposal, vector transformation, and classification. SSP-net [21] optimizes R-CNN in many aspects and improves detection performance. Fast R-CNN [22] combines the essence of R-CNN and SPP-net, and introduces a multi-task loss function, which makes the training and testing of the entire network very convenient. Faster R-CNN [23] uses RPN to replace the selective search module in Fast R-CNN, and RPN shares functions with Fast R-CNN. This greatly improves the time and accuracy of object detection.

Other object detection frameworks do not require the process of generating candidate boxes. The one-stage algorithm can directly extract features from the network to predict object classification and location. The OverFeat method [24] classifies the detection area by sliding windows with different proportions at each feature point of the topmost feature layer. The YOLO method [25] classifies and locates objects in one step, and directly returns the position and category of the bounding box in the output layer. The SSD method [26] uses convolutional kernels on feature maps to predict the class and coordinate offsets of a series of default bounding boxes.

Whether it is a one-stage or two-stage algorithm, they both use anchors extensively. Usually the former uses RPN to generate anchors, and the latter directly classifies and regresses anchors. Therefore, the number and shape of anchors greatly affect the performance of the object detection algorithm. Wang et al. [14] proposed an anchor that can generate sparse and arbitrary shapes through image features, which can reduce the number of anchors and optimize their shape while ensuring a certain recall rate. Cheng et al. [27] proposed a proposal generation method, which can generate more proposals that have higher intersection over union (IoU) with ground truth boxes than those obtained by greedy search approaches, which can better envelop entire objects. Zhang et al. [28] proposed a fast matching algorithm that robustly matches region proposals with massive exemplars in terms of appearance and spatial context, and can robustly handle noisy localizations of image exemplars. Compared with two-stage algorithms in terms of detection speed, one-stage algorithms are faster, but in terms of detection accuracy, the popular version of the former is better than the latter.

The accuracy of detection and classification in the object detection process usually depends on the feature expression of the detection object, which includes two aspects: the feature expression of the area where the detection object belongs, and the feature expression outside the detection area, which involves the fusion of contextual information. Whether or not the object can be characterized well, this tests the detection performance of the algorithm. However, small object detection has problems, such as blurred images, low information, and easy misjudgment. Therefore, the feature expression of small object detection is particularly important. Because the RGB color space is unstable and unreliable, manual features that improve detection efficiency by transforming the original image features [29] were widely used in the initial stage of object detection. Due to the large amount of hand-made feature data and the lack of high-level semantic expression capability, it was gradually replaced by related deep learning technologies.

The R-CNN method [6] uses trained CNNs to classify the object area and then judges whether it belongs to the object or background area. Faster R-CNN [9] uses a VGG16-based network as a feature extraction network and has obtained very good results. Since many methods only focus on detecting the object itself [30,31], the relationship between the object and its surrounding environment is often ignored, and this information is usually helpful for the correct judgment of the detection algorithm. Context-based object detection was summarized, experimented on, and analyzed in [32]. They concluded that the combination of contextual information and object detection can improve detection performance. The proposed inside-outside network (ION) [17] combines contextual information with regions of interest. It uses skip pooling and contextual information with spatial recurrent neural networks, which can detect small and occluded objects. In [33], a multi-scale spatial context was attached to a region-based CNN model to extract the relationship between the object and the background. Multi-scale context was used in [34] to attach to the detection function and improve the detection performance. These methods achieved good results in the performance of object detection, such as accuracy and speed.

Recently, more studies have improved object detection algorithms by using multi-layer and multi-scale representation methods. Compared with using only one layer of deep convolution features, multi-layer and multi-scale feature fusion methods can achieve richer feature expression [35,36,37,38]. A three-layer deep convolution feature in ResNet-50 was used in [39] for small object detection in optical remote sensing images. A feature pyramid network (FPN) was proposed in [40], which used a multi-scale feature fusion method that combined semantic information and location information, greatly improving the performance of object detection, especially for small objects. A backbone network specifically for small object detection was proposed in [41]. This network was an improved algorithm based on the ResNet-50 network, and its detection accuracy was several percentage points higher. Multi-scale and multi-level deep feature information of a fully convolutional neural network was used in [38], which provided more semantic information. Skip layer connection was used to extract features in [42]. The information obtained was especially important for small objects. The results showed that multi-scale representation can improve small object detection.

## 3. SC-Faster R-CNN

Faster R-CNN has significant detection performance for object detection in general scenes, but usually the detected objects in such scenes have problems such as occlusion, deformation, and large scale. In scenes where these special problems exist, the detection performance of Faster R-CNN has greater limitations. The SC-Faster R-CNN algorithm proposed in this paper effectively solves such problems.

**Figure 1:**
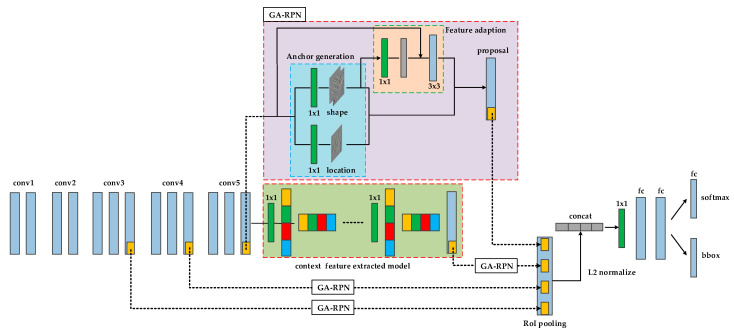
Structural framework of SC-Faster region-based convolutional neural network (R-CNN). The method is the Faster R-CNN framework combined with skip pooling and fusion of contextual information.

The deep network algorithm is based on Faster R-CNN as the framework. The structural framework of SC-Faster R-CNN is shown in Figure 1, and its operation process is as follows:

1. Skip pooling operations are performed on multiple feature layers. Through GA-RPN processing of conv3_3, conv4_3, and conv5_3 multi-feature layers to obtain the feature map mapped to the region of interest (ROI), the size of the mapped area is adjusted to the same size sections, then the max pooling operation is performed on each section, so that fixed-size feature maps can be obtained from ROIs of different sizes.
2. The feature maps are linearly combined by L2 normalize. Because the features between dimensions have certain differences in position and value, L2 normalize can improve the accuracy of the model and make features of the same type in different dimensions have a certain degree of similarity.
3. By using the concat [43] method and 1x1 conv processing, the feature map after ROI mapping is shaped and dimension reduced. The ROI in the processed image will generate a feature vector of a fixed size.
4. The feature vectors are input to the classifier through the fully connected layer to classify the features.

### 3.1. Context Feature Extracted Model

Occlusion is currently a problem that needs to be solved urgently in object detection, because after the object is occluded, its feature information will be greatly reduced, and this will have an effect on the occluded object, making it extremely difficult to detect and easy to be misrecognized [44]. In view of this situation, this paper proposes a feature extraction model based on contextual information. Before introducing the model, a more important theoretical method used by the model is introduced, recurrent neural network (RNN) [45].

**Figure 2:**
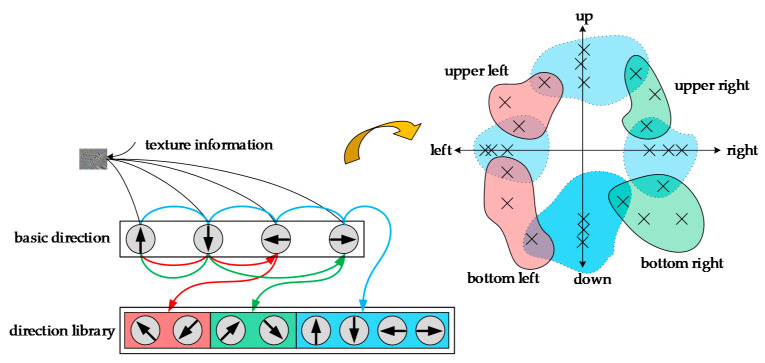
Schematic of recurrent neural network based on directional characteristics in texture features.

From Figure 2, we can see that the recurrent neural network maps all directional features into a two-dimensional vector space. The directions of some of the upper left and lower left features are very close, so the two vectors representing them are very close in the vector space. The upper right and lower left direction features are quite different, and the distance between them is much larger compared to the vectors in the other directions. In this way, by comparing the distance between vectors, a representation of semantic information can be obtained.

**Figure 3:**
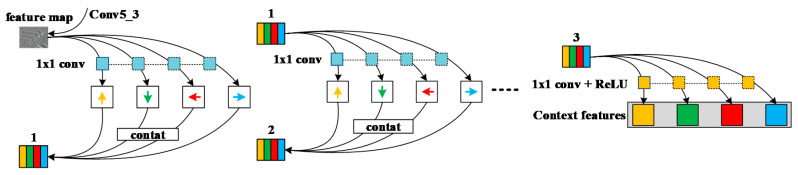
Feature extraction model based on contextual information.

We placed one-way moving RNNs in the last layer of the fifth convolutional layer based on VGG-16 (i.e., conv5_3), as shown in Figure 3. Generally, RNNs will have a certain input loss in each step of the operation, but important details in the image will still learn the feature information well and then output the feature map with contextual information after updating the hidden layer state information. The specific operation is to place the RNNs along the horizontal and vertical directions of the output image of conv5_3, so it can be considered that the RNNs move in four directions on the image: up, down, left, and right. Since initializing the recursive weight matrix as an identity matrix can greatly optimize model training and facilitates good performance in modeling the dependencies of further layers, RNNs adopt the nonlinear method of recursive weight matrix and use it in each step. The image generated in this way has the same size as the output image of conv5_3. In order to strengthen the learning of feature information but also reduce the input loss due to RNN operations, experiments have shown that RNNs can obtain better feature learning results through three types of recursive contextual information learning. This paper uses this structure because it is easy to achieve parallelization, which is conducive to improving network performance.

In order to initialize the weight identity matrix for RNNs, the activation function uses the rectified linear unit (ReLU) function, which can improve the training speed. Le et al. [46] called the motion module composed of ReLU and RNNs as “IRNNs”. There are four IRNNs moving in different directions on the image being operated, and all the IRNNs can obtain information characteristics in the corresponding direction. From the structure diagram of the contextual feature extraction model, it can be seen that before each IRNN operation, it must be filtered by a 1 × 1 shared convolution kernel. The shared convolution kernel can work in different directions, and it can ensure accuracy. In the case of reducing redundant convolution kernels, in the process of sharing, the deviation of each layer will be transmitted with the shared convolution kernel, which helps the effective convergence of the function. The output of IRNNs is based on cascading calculations of the hidden states in different directions at each spatial position.

When IRNNs move from left to right or top to bottom in the image, each time an input is consumed, a hidden unit is updated and an output is generated at the same time. In this way, it expands in four directions from left to right, right to left, top to bottom, and bottom to top. In order to realize the function of IRNNs as efficiently as possible, the internal IRNNs are split and calculated during the operation. The formula of IRNNs for different directions in the process of moving is

(1) $$f_{i,j}^{derc}\leftarrow \max\left(W_{ff}^{derc}f_{i,j-1}^{derc}+f_{i,j}^{derc},0\right)$$

where *derc* represents the four motion directions of IRNNs (up, down, left, and right); $W_{ff}^{derc}$ and $f_{i,j-1}^{derc}$ represent the weight of the hidden state in the corresponding direction and the input from 1×1 convolution to the hidden layer, respectively. In order to simplify the calculation, set $W_{ff}^{derc}$ as the identity matrix, so that Formula (1) is equivalent to an accumulator:

(2) $$f_{i,j}^{derc}\leftarrow \max\left(f_{i,j-1}^{derc}+f_{i,j}^{derc},0\right).$$

### 3.2. GA-RPN

Anchors are widely used in object detection based on deep learning as a mechanism for generating object region proposals. The number and shape of anchors greatly affect the performance of the object detection algorithm. Since the commonly used anchors have a limited aspect ratio, in order to maintain a high recall rate, more anchors need to be generated, which is not only inefficient but also leads to decreased detection accuracy, because most of the area surrounded by the anchor frame is background. Therefore, an anchor generation strategy that can effectively reduce the number of anchors and tightly surround the object without manual intervention will have great significance for optimizing the performance of object detection.

In this paper, we utilized the guided anchor method instead of the RPN in Faster R-CNN to generate anchors with no redundancy and suitable size. In the guided anchor method, first, we used four parameters (*x, y, w, h*) to describe an anchor, where (*x, y*) represents the coordinates and w and h represent the width and height, respectively, of the anchor. Therefore, the distribution probability of the anchor on image I can be expressed by the formula

(3) p(x,y,w,h|I)=p(x,y|I)p(w,h|x,y,I).

As can be seen from the formula, the probability distribution of the anchor is decomposed into two conditional probability distributions, that of the anchor center point on a given feature map and that of the shape after the image feature and the center point. These two probabilities respectively indicate that the object may have a certain area and the shape of the object has a close relationship with its position.

According to the distribution probability formula of the anchor on image *I*, Wang et al. [14] designed an anchor generation module that uses two branches to predict the position and shape of the anchor. For example, in the skip pooling branch in this paper, we first used the anchor position prediction branch to generate a probability distribution map in feature map F1, which indicates the possible position of the object in the image. Similarly, the shape predicts the shape of the branch position. The results generated by these two prediction branches are combined, and then the most likely object position and corresponding shape are selected through a preset threshold.

#### 3.2.1. Position Prediction of Anchor

**Figure 4:**
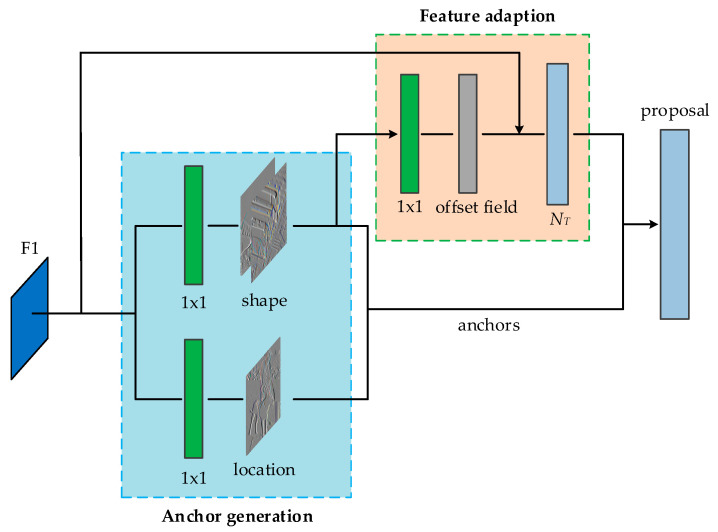
Architecture of guided anchor, based on anchor generation module with two branches to predict the anchor location and shape. Feature adaption module is applied to the feature map to make the new feature map aware of anchor shapes.

As shown in Figure 4, the size of the probability distribution map generated by the anchor position prediction branch is the same as the input feature map F1. The area of the entire feature map is divided into an object center area, a peripheral area, and an ignored area through the position probability distribution map. The main idea is to set a small piece of ground truth boxing center corresponding to the area on F1 as a positive sample during the training process, and set the threshold for the remaining areas to mark the ignore area or peripheral area according to the distance from the center. Through such position prediction, part of the area can be screened out as the candidate center position of the anchor, so that the number of anchors is greatly reduced while still maintaining the same recall rate. When doing inference, because there is no need to consider the excluded area, masked convolution can be used instead of ordinary convolution, so that calculations can be performed only where there is an anchor, thereby speeding up the calculation.

#### 3.2.2. Shape Prediction of Anchor

The purpose of the shape prediction branch is to predict the best length and width given the anchor center point, which is a regression problem. According to the usual practice, first calculate the object, that is, the optimal w and h of the anchor at the center point, and then use losses such as L1, L2, or Smooth L1 to supervise. The length and width of the object are not easy to calculate, and it will be more difficult to implement, so we directly use IoU as supervision to learn *w* and *h*.

Previously, *IoU* was used to directly calculate the anchor and all ground truth, but the current w and h are uncertain, so they cannot be calculated according to the conventional algorithm. To solve this problem, refer to [14], which defines the *IoU* between a variable anchor $a_{wh}\;=\;\left\{\left(x_{0},\;y_{0},\;w,\;h\right)|w\;>\;0,\;h\;>\;0\right\}$ and a ground truth bounding box $gt\;=\;\left(x_{g},\;y_{g},\;w_{g},\;h_{g}\right)$ as follows, denoted as *vIoU*:

(4) $$vIoU(a_{wh},gt)\;=\;\underset{w>0,h>0}{max}IoU_{normal}(a_{wh},gt).$$

Since the parameters in the formula are all variables, the calculation of $vIoU(a_{wh},gt)$ is complicated and it is difficult to realize effective operation in the end-to-end network, so an approximate substitution method is adopted to solve this problem. The approximate replacement method is to match the anchor with the specific *gt* according to the sampled w and h, and then use the bounded *IoU* loss as the loss of this branch.

(5) $$L_{shape}=L_{1}(1-min(\frac{w}{w_{g}},\frac{w_{g}}{w}))\;+L_{1}(1-min(\frac{h}{h_{g}},\frac{h_{g}}{h}))$$

Here, (*w, h*) and (*w_g_, h_g_*) represent the shape of the predicted anchor and the corresponding ground-truth bounding box, and *L*_1_ is the smooth *L*_1_ loss.

#### 3.2.3. Anchor-Guided Feature Adaptation

In different positions of conv in the same layer, the receptive field of the feature is the same. In the original RPN, both represent anchors of the same shape, but now each anchor has its own unique shape and size, which does not match the feature particularly well. In addition, for the original feature map, it does not know the shape of the anchor or the branch prediction, but the subsequent classification and regression are based on the predicted anchor, and errors may occur. So, a feature adaption module was added to solve this problem.

This module directly integrates the shape information of the anchor into the feature map, so that the newly obtained feature map can adapt to the shape of each position anchor. Using a 3 × 3 deformable convolution to correct the original feature map, the offset of the deformable convolution is obtained through a 1 × 1 conv through the *w* and *h* of the anchor. Through this operation, the purpose of making the effective range of the feature closer to the shape of the anchor is achieved, and different positions of the same conv can also represent anchors of different shapes and sizes. The formula of feature adaption is as follows:

(6) $$f_{i}^{\prime }=N_{T}(f_{i},\;w_{i},\;h_{i})$$

where $f_{i}$ is the feature at the *i*th location, (*w_i_, h_i_*) is the corresponding anchor shape, and $N_{T}$ represents a 3 *×* 3 deformable convolutional layer.

### 3.3. Multi-Layer Feature Fusion

The deep neural network algorithm is currently the mainstream method in the field of object detection. It extracts and classifies features in an image through autonomous learning during the training process and finally achieves the effect of object detection. The network structure in deep neural network algorithms is usually divided into shallow and deep networks. The characteristics of shallow network are that it has a relatively small receptive field and a strong ability to represent detailed geometric information. Although the resolution is high, its ability to represent semantic information is very weak [47]. The characteristics of the deep network are that it has a relatively large receptive field and a strong ability to represent semantic information, but the resolution of its feature map is low, and details of spatial geometric feature are lacking [48]. Usually objects of different scales are stored in the scene of object detection at the same time. In order to be able to detect large and small objects at the same time, it is necessary to use the small receptive field network to obtain feature information for small objects and the large receptive field network to process large objects. Therefore, this paper uses a multi-layer feature fusion method to solve this problem.

The fusion of features of different dimensions is actually the superposition of information on the input data, which can enrich the information and improve the performance of the model [49]. For feature fusion that is directly added, the premise is that the dimensions are the same. If the dimensions of the initial features are not the same, then the features of different dimensions need to be linearly combined. The original features are projected to the same dimensional space, then feature fusion is performed. Because the dimensions of feature layers in this paper are different, it is necessary to linearly combine the features of the different dimensions of each layer before feature fusion. The linear combination method used in this paper is L2 normalize [50].

The main idea of normalization is to calculate the p-norm [51] for each sample, then divide each element in the sample by the norm. The result of this processing is to make the *p*-norm of each processed sample equal to 1. The formula is defined as

(7) $${\left|\left|X\right|\right|}_{p}={\left({\left(x_{1}\right)}^{p}+{\left(x_{2}\right)}^{p}+\cdots +{\left(x_{n}\right)}^{p}\right)}^{\frac{1}{p}}.$$

The loss function of L2 regularization is

(8) $$L\left(\omega \right)=L_{D}\left(\omega \right)+\frac{\lambda }{2n}\sum_{i=1}^{n}\omega_{i}^{2}.$$

The gradient of the loss function *L* (*ω*) is calculated by

(9) $$\frac{\partial L\left(\omega \right)}{\partial \omega }=\frac{\partial L_{D}\left(\omega \right)}{\partial \omega }+\lambda \omega .$$

The parameter *ω* is updated to become

(10) $$\omega^{\prime }=\omega -\eta \frac{\partial L\left(\omega \right)}{\partial \omega }=\omega \left(1-\frac{\eta \lambda }{n}\right)-\frac{\partial L_{D}\left(\omega \right)}{\partial \omega }$$

where $L_{D}\left(\omega \right)$ represents the sum of squares of the difference between the object value and the predicted value; $\frac{\lambda }{2n}\sum_{i=1}^{n}\omega_{i}^{2}$ represents a penalty item; n is the number of object categories in the feature layer; and λ, $\omega_{i}$, and i are the weight coefficient, the weight coefficient of various objects, and the index of a certain category of objects, respectively. According to $\omega^{\prime }$, we know that the parameters after regularization update are one more $\frac{\eta \lambda }{n}\omega$ than those without updates. When $\omega^{\prime }$ tends toward 0, the speed of parameter decrease becomes very slow. Therefore, the parameters can be reduced to a small range, so that the cosine similarity of two vectors can be obtained, and the features of different layers can be projected to the same dimensional space. This facilitates better feature fusion for subsequent feature maps of different scales from the same figure.

## 4. Analysis of Experimental Results

In order to reflect the high detection performance of SC-Faster R-CNN, first, it should be reflected in the improved module based on Faster R-CNN, which has relatively obvious improvement in the detection performance of certain features of the object. Therefore, it is necessary to start the experiment of the influence of the improved module on the performance of the entire network one by one, that is, the ablation experiment of the proposed method. Then, SC-Faster R-CNN is compared with Faster R-CNN and other related algorithms in the same field. Through such a series of comparative experiments, we can evaluate the comprehensive performance of SC-Faster R-CNN in the object detection process. We evaluated the detection performance of these algorithms on the PASCAL VOC 2007 and 2012 datasets for experimental verification. Three experiments were done: selection of the number of groups of IRNNs and the feature layers, ablation of SC-Faster R-CNN, and comparison experiment of SC-Faster R-CNN with other algorithms.

### 4.1. Experimental Platform Construction

The experimental implementation platform of this paper is 64-bit Ubuntu 16.04 LTS based on a Dell Precision R7910 (AWR7910) graphics workstation, the processor is Intel Xeon e5-2603 v2 (1.8 gHz/10 M), and Quadro K620 GPU is used for accelerated calculation. As a framework for deep neural network algorithm training, Pytorch takes into account the calculation speed and modularity. Training in the Pytorch framework has faster computing speed thanks to its support for parallel computing on CPU and GPU.

#### 4.1.1. Network Training Parameter Settings

The initial learning rate of the training model was 0.01, the optimization method used stochastic gradient descent, the gamma value was 0.1, the momentum was 0.9, the weight decay was 0.005, the maximum number of iterations was 80,000, the learning rate for the first 20,000 times was 0.01, and the learning rate for the next 60,000 times was 0.001.

#### 4.1.2. Dataset

The experiment was carried out on the benchmark PASCAL VOC detection dataset, which includes the PASCAL VOC 2007 and 2012 datasets (represented as 07 + 12). These two datasets have 9963 and 23,080 images for the object detection task, respectively, including 116,492 test images. The test images contain 39,482 objects. During the experiment, 16,551 images from the 2007 and 2012 train + val datasets were used for training, and 4952 images from the 2007 test dataset were used for testing.

#### 4.1.3. Evaluation Index and Loss Function

In order to evaluate the detection performance of deep neural network algorithms, this paper uses several evaluation indicators: precision, recall rate, intersection over union (IoU), mean precision (mP), and mean average precision (mAP). The evaluation standard judges the pros and cons of the corresponding attribute according to the value of the evaluation index. In these evaluation indices, the larger the value, the better the corresponding attribute. The formulas for accuracy, recall, IoU, and mAP are defined as follows:

Precision:

(11) $$\mathrm{Precision}=\frac{T_{P}+T_{N}}{T_{P}+T_{N}+F_{P}+F_{N}}\times 100\%$$

Recall:

(12) $$\mathrm{Recall}=\frac{T_{P}}{T_{P}+T_{N}}\times 100\%$$

IoU:

(13) $$\mathrm{IoU}=\frac{GT\cap DR}{GT\cup DR}\times 100\%$$

mAP:

(14) $$\mathrm{mAP}=\overset{N}{\underset{k=1}{\sum }}P\left(k\right)\Delta r\left(k\right)$$

where $T_{P}$, $T_{N}$, $F_{P}$,$\;\mathrm{and}\;F_{N}$ are real objects predicted as real objects, false objects predicted as real objects, real objects predicted as false objects, and false objects predicted as false objects, respectively; *GT* is the ground truth of object boxes; *DR* is the test result; *N* is the number of figures in the test set; *P(k)* represents the value of precision when k figures can be recognized; and Δr(k) represents the change of the recall value when the number of recognized figures changes from *k* − 1 to *k*.

For the images in the training dataset, the model will aim to minimize the joint loss during the training process and fine-tune the network parameters. The loss function is defined as

(15) $$L=\frac{1}{N_{c}}\underset{k}{\sum }L_{c}\left(P_{k},P_{k}^{*}\right)+\frac{\lambda }{N_{r}}P_{k}^{*}L_{t}\left(t_{k},t_{k}^{*}\right)$$

where *L*, $L_{c}$, and $L_{t}$ are the joint loss, classification loss, and regression loss of the border; $N_{c}$ and $N_{r}$ are the number of categories and boxes; λ and *k* represent the weight coefficient and the selected anchor box index; $P_{k}$ and $P_{k}^{*}$ represent the probability that candidate box k is the object, the value of the label (if the candidate box is a positive label, $P_{k}^{*}=1$, otherwise $P_{k}^{*}=0$); $t_{k}^{*}$ is the predicted offset of the anchor box; and $t_{k}^{*}$ is the offset between the anchor box and the actual object box.

### 4.2. Parameter Selection Experiment of Detection Algorithms

Before conducting comparative experiments on multiple object detection algorithms, first we discuss the context feature extraction module and skip pooling method.

For the context feature extraction module, the number of groups of RNNs will affect the detection performance and efficiency of the network. In order to effectively carry out comparative experiments, the impact of the number of groups of RNNs on the detection performance has to be considered. In order to facilitate the experiment, for the training framework we chose RS-Faster R-CNN, for which the detection performance is evaluated by adjusting the number of IRNN groups to select the optimal number.

As shown in Table 1, the number of IRNNs in the experiment ranged from 1 to 5. In order to obtain the optimal context feature extraction module, the evaluation standard was mAP. It can be seen that when the number of IRNNs increases from 1 to 3, the value of mAP increases significantly, and the data become flatter when it reaches 4 and 5. In order to ensure the detection accuracy of the object detection algorithm while considering the calculation efficiency, the number of IRRN groups was selected as 3.

For the selection of multi-layer features in the skip pooling method, it is necessary to discuss which feature layers should be selected and the effect of post-processing them on the detection performance of SC-Faster R-CNN. This paper discusses the feature layers that may become skip pooling operations one by one, as shown in Table 2. It can be seen that the presence or absence of L2 normalize has a great impact on the accuracy of detection. If there is no L2 normalize processing, the accuracy will decrease rapidly as feature layers increase. Through the L2 + 1 × 1 item in the last three rows of the “Merge features using” column, it can be found that the feature layer of skip pooling operation includes either C2 or both C1 and C2, which slightly promotes accuracy. Compared to C3 + C4 + C5+context, it brings a lot of calculation to the deep neural network algorithm. Based on this situation, C3 + C4 + C5+context was chosen as the feature layer of skip pooling operation.

### 4.3. Ablation Experiment of SC-Faster R-CNN

In order to evaluate whether the context feature extraction module and skip pooling method were added to the improved Faster R-CNN and how the detection performance is affected, the ablation experiment on SC-Faster R-CNN was conducted. First, the network after removing the module on the framework is referred to as RC-Faster R-CNN; the network without the skip pooling method on the framework is referred to as RS-Faster R-CNN; and SC-Faster R*-CNN recommends reducing GA-RPN to RPN for the proposed method. Then, the object detection performance of RC-Faster CNN (RC-FR), RS-Faster R-CNN (RS-FR), SC-Faster R*-CNN (SC-FR*), and SC-Faster R-CNN (SC-FR) was compared with Faster R-CNN (FR).

Table 3 shows the main performance parameters of the five deep neural networks in ablation experiments. By analyzing the values of mAP, it can be found that based on the method proposed in this paper, the effect of the context feature extraction module and skip pooling method acting alone on the 07 + 12 dataset is slightly better than the detection effect of Faster R-CNN. The time consumption of RS-Faster R-CNN and RC-Faster R-CNN in a figure is slightly larger than Faster R-CNN, however, the detection accuracy is superior. It is worth noting that after the regional recommendation method is changed from RPN to GA-RPN, the detection accuracy and operation speed of the proposed method in this paper are improved. In terms of test time, SC-Faster R-CNN is not only faster than Faster R-CNN, but also it has great advantages in detection performance improvement.

**Figure 5:**
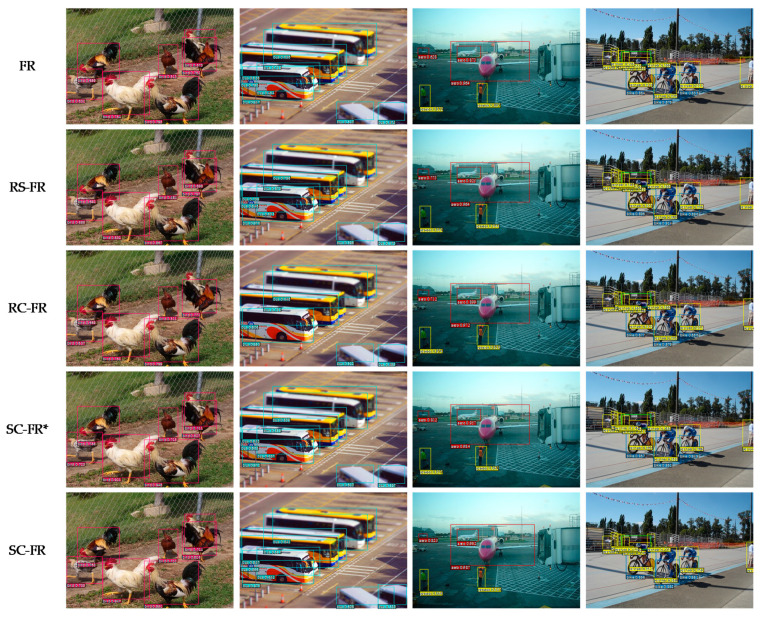
Some examples of detection results of the proposed algorithm ablation experiment.

Figure 5 shows part of the experimental test results, which can be found intuitively by combining the test results in Table 3. RS-Faster R-CNN and RC-Faster R-CNN under different scales and degrees of occlusion and using different detection algorithms have their own outstanding detection performance for certain features. Combining the evaluation parameter mAP (%) and the test time, it can be found that Faster R-CNN has greatly improved detection performance after adding the context feature extraction module and skip pooling method. Therefore, the detection performance of these five detection algorithms under different conditions can be ranked:

(1) Comparison of detection accuracy:
  - When detecting small objects:
    SC-FR>SC-FR*>RC-FR>RS-FR>FR
  - When part of the object is occluded:
    SC-FR>SC-FR*>RS-FR>RC-FR>FR
(2) Comparison of detection efficiency:
  FR>RC-FR>RS-FR>SC-FR>SC-FR*

### 4.4. Comparative Experiment of Multiple Detection Algorithms

In this section, we present comparison experiments on the detection performance of the five networks: SC-Faster R-CNN with Faster R-CNN [23], Multi-Scale CNN(MS-CNN) [52], HyperNet [42], SSD512 [26], and YOLOv3 [53]. The comparison experiment mainly focuses on the detection accuracy and calculation efficiency of each algorithm.

#### 4.4.1. Accuracy

**Figure 6:**
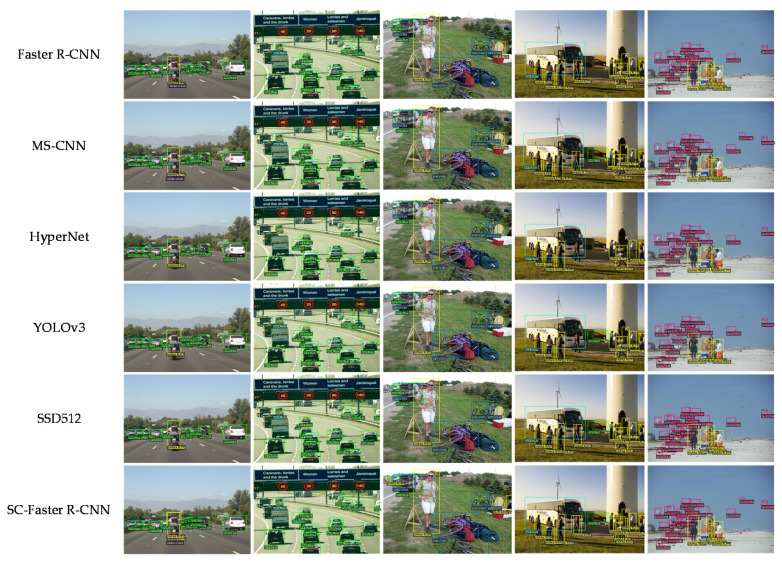
Proposed algorithm and comparison algorithms on detection effect of different sizes of objects in 07 + 12 dataset.

Table 4 shows the detection results of different networks on the 07 + 12 dataset and ranks the detection accuracy according to the magnitude of the mAP value in the table: SC-Faster R-CNN > SSD512 > MS-CNN > HyperNet > YOLOv3 > Faster R-CNN. Some examples of detection effects for different networks are depicted in Figure 6 for the 07 + 12 dataset.

**Figure 7:**
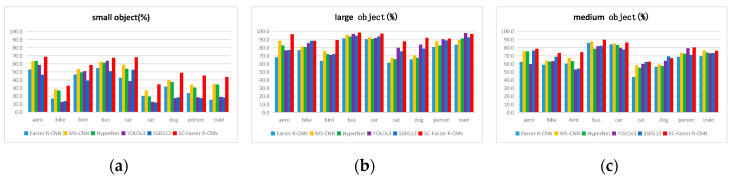
Detection of (**a**) small, (**b**) medium, and (**c**) large objects in test set of 07 + 12 dataset by various deep neural network algorithms.

Figure 7, with an mP evaluation index, shows the detection of small, medium, and large objects in the test set of the 07 + 12 dataset by various detection algorithms, that is, the detection results of different types and scale objects in the test set by each detection algorithm. Figure 7, especially Figure 7a, shows HyperNet, an algorithm for feature fusion from multiple feature layers, and MS-CNN, the multi-scale detection algorithm and the method proposed in this paper. Their comprehensive detection performance for small object detection is better than other algorithms and other networks. With the cooperation of the context feature extraction module and the skip pooling method, the detection performance of SC-Faster R-CNN for small objects was greatly improved on the basis of Faster R-CNN. It can be seen in Figure 7b,c that as the object scale becomes larger, the detection performance of each algorithm gradually tends to approximate.

**Figure 8:**
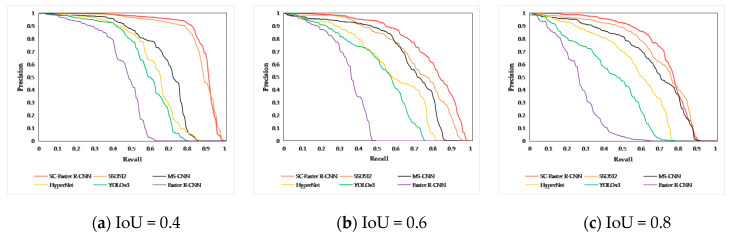
PR curve images of various deep neural network algorithms. The object detection situation for different networks in the 07 + 12 dataset can be seen more intuitively when the threshold of intersection over union (IoU) is different: (**a**) when IoU = 0.4, the overall detection performance of each network is not much different, but SC-Faster R-CNN is better than other networks; (**b**) when IoU = 0.6, the detection performance of most networks drops sharply, but that of SC-Faster R-CNN is stable; (**c**) when IoU = 0.8, the overall detection performance of each network decreases, but SC-Faster R-CNN still has splendid detection performance compared to other networks.

In order to more intuitively reflect the detection performance of SC-Faster R-CNN, this paper discusses that when the threshold of IoU is 0.4, 0.6, and 0.8, various detection algorithms are based on the 07 + 12 dataset to obtain precision-recall (PR) curve images. According to Figure 8, it can be concluded that with increased IoU threshold, the improved SC-Faster R-CNN based on the Faster R-CNN framework highlights the state-of-the-art detection performance. The results of the PR curve images are consistent with the conclusion of Table 4.

#### 4.4.2. Calculation Efficiency

In the training and calculation process of deep neural network algorithms, hundreds of millions of parameters are often involved. The calculation of these parameters requires a lot of computing power. The deep neural network algorithms involved in the object detection experiment in this paper all run on GPU. An excellent object detection algorithm not only needs to focus on detection accuracy, but also consider detection efficiency. In many fields, such as unmanned driving and military object intrusion, the efficiency of object detection has extremely strict requirements. This section discusses the important performance indicators when testing on the 07 + 12 dataset.

It can be seen from Table 5 that the calculation speed of the one-stage algorithm is much higher than that of the two-stage algorithm, but according to the above experimental results, the one-stage algorithm needs to weigh the detection accuracy and time-consumption issues in the object detection process. By comparing SC-Faster R-CNN and SC-Faster R*-CNN, it can be seen that the former’s area recommendation method is improved from RPN to GA-RPN, therefore, the number of proposals is greatly reduced, so both mAP and FPS have been improved.

## 5. Conclusions and Future Work

This paper introduces the Faster R-CNN algorithm with skip pooling and fusion of contextual information (SC-Faster R-CNN). The algorithm is based on the Faster R-CNN framework and adds a feature extraction model and skip pooling method that integrates contextual information and uses guided anchors with high performance instead of RPN. In order to prove that it has high detection performance, we conducted ablation and comparison experiments between the proposed algorithm and Faster R-CNN, YOLOv3, SSD512, HyperNet, and MS-CNN. Training and testing on PASCAL VOC 2007 and 2012 datasets produced state-of-the-art results. Through experimental results, it is found that the algorithm proposed in this paper is particularly effective for difficult problems in the detection of partially occluded and small objects.

In this paper, the proposed algorithm has high detection performance in general and for objects that are too small or partially occluded, but it still has certain limitations in the detection of deformed, rotating, and camouflaged objects. In order to further improve the detection efficiency, we will pay more attention to the research of difficult sample detection. In order to meet the real-time performance of the system, we will further study the processing speed of the algorithm and other issues. Also, researching an object detection algorithm with strong generalization ability but considerable computational efficiency will be a meaningful future research direction.

## Figures and Tables

**Table 1:** Influence of number of IRNNs groups in context feature extraction module on object detection accuracy.

mAP (%)	Time (sec)	Number of IRNNs				
		1	2	3	4	5
70.5	0.58	√				
72.2	0.71	√	√			
76.7	0.79	√	√	√		
76.9	0.84	√	√	√	√	
77.1	1.63	√	√	√	√	√

**Table 2:** Effect of combining features from different layers on detection accuracy.

ROI Pooling From						Merge Features Using			
C1	C2	C3	C4	C5	Context	1 × 1		L2 + 1 × 1	
						mAP	Time	mAP	Time
					√	68.4	0.72 s	70.9	0.73 s
				√	√	66.1	0.76 s	73.8	0.76 s
			√	√	√	62.5	0.80 s	75.3	0.79 s
		√	√	√	√	58.6	0.82 s	77.8	0.81 s
	√	√	√	√	√	52.7	0.91 s	78.0	0.88 s
√	√	√	√	√	√	44.3	1.45 s	78.4	1.17 s

**Table 3:** Main performance parameters in ablation process of SC-Faster R-CNN.

Method	Train	mAP (%)	Time (sec)
FR	07 + 12	70.8	2.2
RS-FR	07 + 12	73.2	1.1
RC-FR	07 + 12	71.7	0.6
SC-FR*	07 + 12	75.8	2.9
SC-FR	07 + 12	77.6	0.8

**Table 4:** Result of PASCAL VOC 2007 test set (%). Training data: 07 + 12: VOC 2007 and 2012 train + val.

Method	Train	mAP	Aero	Bike	Bird	Boat	Bottles	Bus	Bar	Bat	Chair	Bow	Table	Dog	Horse	mbike	Person	Plant	Sheep	Sofa	Train	TV
Faster R-CNN	07 + 12	70.8	83.1	71.8	73.2	53.5	54.4	77.3	72.8	88.4	49.2	72.7	66.1	85.4	80.3	79.6	74	43.2	78.8	67.4	81.5	63.4
MS-CNN	07 + 12	73.2	79.8	78.6	74.7	59.1	54.8	77.2	73.6	90.3	61.3	77.3	65.7	81.2	84.7	83.6	76.9	50.7	72.6	67.5	83.4	70.9
HyperNet	07 + 12	71.2	81.7	70.4	67.2	55.7	51.7	78.6	79.9	88.9	52.4	74.3	63.6	84.3	81.7	80.7	79.1	47.3	71.2	64.2	80.2	71.7
YOLOv3	07 + 12	72.0	80.4	75.3	70.9	56.8	53.3	79.6	75.7	86.7	58.8	75.6	60.9	79.4	82.6	81.5	79.3	52.3	71.5	65.6	84.8	68.9
SSD512	07 + 12	74.2	84.8	73.5	74.3	57.3	57.6	80.5	74.8	89.5	60.2	82.1	68.4	86.3	84.5	81.4	77.6	49.3	82.4	66.5	79.7	72.8
SC-Faster R-CNN	07 + 12	77.6	86.5	82.4	76.4	61.4	60.5	86.5	76.2	92.1	64.5	83.6	69.3	90.1	89.4	88.5	79.2	58.2	79.1	70.2	85.6	72.7

**Table 5:** Important performance indicators of each algorithm when testing on 07 + 12 dataset.

Method	mAP	FPS	Batch Size	Proposals
Faster R-CNN	70.8	5	1	2460
MS-CNN	73.2	8	1	548
HyperNet	71.2	5	1	1140
YOLOv3	72.0	87	1	98
SSD512	74.2	56	8	24,564
SC-Faster R*-CNN	75.8	5	1	2460
SC-Faster R-CNN	77.6	9	1	473
